# Reconstructing Liver Fibrosis: 3D Human Models, Microbiome Interfaces, and Therapeutic Innovation

**DOI:** 10.3390/cimb48020165

**Published:** 2026-02-01

**Authors:** Dileep G. Nair, Divya B. Nair, Ralf Weiskirchen

**Affiliations:** 1 Institute of Molecular Pathobiochemistry, Experimental Gene Therapy and Clinical Chemistry (IFMPEGKC), RWTH University Hospital Aachen, D-52074 Aachen, Germany; 2 LivMira Therapeutics Inc., San Diego, CA 92129, USA

**Keywords:** liver fibrosis, extracellular matrix, metabolic dysfunction-associated steatotic liver disease, cirrhosis, metabolic dysfunction-associated steatohepatitis

## Abstract

Liver fibrosis is a significant consequence of severe liver injury resulting from viral hepatitis, alcohol, and metabolic dysfunction. Progressive fibrosis and ultimate cirrhosis are leading causes of morbidity and mortality worldwide, generally irreversible and poorly targeted by current therapies. Traditional in vitro models and animal models mostly fail to fully recapitulate human multicellular crosstalk, extracellular matrix (ECM) remodeling, and the chronic, immune modulated nature of the disease. Recent advances in three-dimensional (3D) cell culture models including organoids, spheroids, bioprinted constructs, and organ-on-a-chip systems are advantageous for reconstructing cellular diversity and mechanical microenvironments to understand pathophysiology and aid in drug discovery. Emerging multi-organ models are capable of incorporating microbiome derived cues and using multi-omics readouts and imaging-enabled mechanistic dissection for more predictive anti-fibrotic screening. These technologies align well with the recent Modernization 3.0 regulation and New Approach Methodologies by the Food and Drug Administration (FDA) and recent EU Pharmaceutical Reform. This review summarizes the pathophysiology of liver fibrosis, the current landscape of 3D human liver models, and examines how microbiome interfaces modulate fibrogenesis.

## 1. Introduction

Liver fibrosis is a pathological consequence of chronic hepatic injury, characterized by the excessive or disproportionate deposition of extracellular matrix (ECM) proteins. The main causes of hepatic fibrosis include persistent injury from viral infections (hepatitis B and C), alcoholic liver disease (ALD), metabolic dysfunction-associated steatotic liver disease (MASLD), metabolic dysfunction-associated steatohepatitis (MASH), autoimmune hepatitis, primary sclerosing cholangitis (PSC), and primary biliary cholangitis [1]. If left unresolved, fibrosis can progress to cirrhosis, a state of extensive architectural distortion and impaired hepatic function. Cirrhosis increases the risk of end-stage liver disease and hepatocellular carcinoma (HCC), with liver transplantation being the only curative option. Currently, there are no approved anti-fibrotic agents for liver fibrosis due to gaps in mechanistic understanding and limitations of traditional preclinical models. Recently approved therapies, such as Rezdiffra^TM^ (Madrigal, West Conshohocken, PA, USA) and Wegovy ^®^ (Novo Nordisk, Copenhagen, Denmark), manage symptoms [2]. Rezdiffra^TM^ acts as a selective thyroid hormone receptor-beta (THR-β) agonist in the liver, improving lipid metabolism and reducing inflammation, while Wegovy^®^ is a GLP-1 receptor agonist that regulates appetite, delays gastric emptying, enhances insulin secretion, and reduces glucagon release to support weight loss and metabolic control [3]. Advancements in 3D models, artificial intelligence (AI) platforms, and linking microbiome research can have a critical impact on developing reversible liver fibrosis therapies [4]. However, further research and development activities are needed to address the complexity of establishing ex vivo models, including reproducibility, vascularization, long term culture stability, and inter-individual microbiome variability. Additionally, with rapidly changing regulatory pathways, standardized validation metrics, community benchmarks, and a roadmap for model qualification within preclinical drug development pipelines are crucial.

## 2. Pathophysiology of Liver Fibrosis

### 2.1. Core Cellular Players

**Figure 1:**
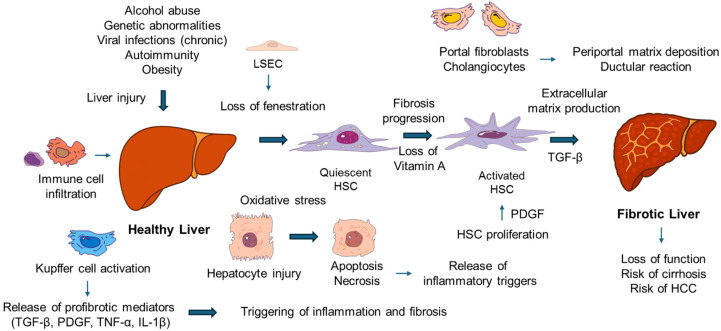
Liver fibrogenesis during liver injury through activation of hepatic stellate cells (HSCs). This schematic figure illustrates the common causes and progression of fibrosis. Abbreviations used: IL-1β, interleukin-1β; LSEC, liver sinusoidal liver cells; PDGF, platelet-derived growth factor; TGF-β, transforming growth factor-β; TNF-α, tumor necrosis factor-α. For more information, refer to the text.

Inflammation caused by liver damage can promote fibrogenesis leading to cellular damage. Liver fibrosis is a dynamic wound healing response involving various cell types such as hepatocytes, hepatic stellate cells (HSCs), liver sinusoidal endothelial cells (LSECs), Kupffer cells, infiltrating monocyte-derived macrophages, cholangiocytes, and other stromal elements [5]. Hepatocyte injury from toxins, lipotoxicity, infections, or cholestasis results in the release of proinflammatory mediators, which activate resident macrophages and recruit circulating immune cells (Figure 1). These immune cells then secrete profibrogenic cytokines like transforming growth factor-β (TGF-β), platelet-derived growth factor (PDGF), and interleukins, leading to the modulation of the ECM composition and excessive buildup of ECM proteins during fibrogenesis. Major triggers that activate HSCs include oxidative stress, inflammation, and growth factors [6]. Cellular changes during fibrogenesis involve the transdifferentiation of quiescent, vitamin A-storing HSCs into proliferative, contractile myofibroblasts that produce fibrillar collagens (type I and III), fibronectin, and other ECM components.

LSECs also play a role in fibrogenesis by losing their fenestrations and adopting a capillarized phenotype, which alters intrahepatic hemodynamics and paracrine signaling to HSCs. Cholangiocytes and portal fibroblasts are particularly significant in cholestatic liver diseases, contributing to periportal matrix deposition and ductular reaction. The interaction among these diverse cell types, mediated by soluble factors, exosomes, and ECM, influences the spatial and temporal progression of fibrotic lesions.

A defining feature of liver fibrosis is the reciprocal communication between hepatic stellate cells and the immune compartment, which collectively shapes both the progression of scarring and its potential reversal [1]. Activated macrophages, including resident Kupffer cells and infiltrating monocyte-derived populations release profibrotic mediators such as TGF-β, PDGF, TNF-α, and IL-1β that support HSC proliferation, survival, and matrix production. In turn, chemokines and growth factors produced by activated stellate cells influence macrophage recruitment and polarization, reinforcing this bidirectional loop [7]. Natural killer cells (NK) add an additional layer of regulation by recognizing and eliminating activated stellate cells through NKG2D ligands and TNF-related apoptosis-inducing ligand (TRAIL), an anti-fibrotic mechanism that is weakened when NK cells become dysfunctional or exhausted. Adaptive immune subsets, including regulatory T- cell, further modulate stellate-cell activity through cytokines such as IL-10 and TGF-β, thereby shaping the balance between inflammatory and reparative responses. As fibrosis regresses, macrophages adopt restorative phenotypes characterized by matrix-degrading enzyme expression and clearance of apoptotic stellate cells, underscoring how coordinated immune–stromal interactions govern the dynamic course of liver fibrosis.

### 2.2. Key Pathways

Multiple signaling pathways result in HSC activation and matrix remodeling, with TGF-β/SMAD signaling representing a central pathway for transcriptional induction of collagen and tissue inhibitors of metalloproteinases (TIMPs) [1,2,3]. The TGF-β superfamily comprises TGF-βs, bone morphogenetic proteins (BMPs), growth differentiation factors (GDFs), activins, and nodal factors, with TGF-β being the major player. The TGF-β family consists of three primary isoforms, TGF-β1, TGF-β2, and TGF-β3, which are involved in fundamental physiological processes ranging from embryogenesis and organ development to tissue repair. TGF-β functions as a master regulator of immune tolerance, inhibiting T-cell proliferation, fostering regulatory T-cell (Treg) generation, and modulating T helper (Th1)/T helper 17 (Th17) differentiation, thereby influencing outcomes in autoimmunity and tumor immunity. Expression of this cytokine is driven by diverse stimuli, including oxidative stress, proinflammatory cytokines, and Toll-like receptor (TLR) signaling, alongside interactions with the ECM and MMPs. TGF-β is produced by macrophages, epithelial cells, platelets, fibroblasts, and T cell, serving as a key activator of fibroblasts and playing a significant role in fibrogenesis.

The PDGF family comprises four isoforms and exerts their biological activity through tyrosine kinase receptors. Their roles in HSC and portal fibroblast proliferation, migration and survival are well studied [8]. Similarly, the expression levels of the PDGF ligand and receptors are increased under liver fibrosis conditions, and blockage of the pathway using therapeutic antibodies and the usage of dominant negative soluble PDGF receptors have been shown to reduce hepatic fibrogenesis [9].

The roles of the ECM, apart from providing the scaffold, include interacting with the cells and affecting pathophysiological conditions. Integrins and other mechanosensors like Piezo 1 translate the changes in ECM composition and stiffness into intracellular signals that affect the myofibroblast phenotype [10]. Ways to modulate integrins can be a means to control fibrogenesis. ECM crosslinking enzymes, such as lysyl oxidase (LOX) and LOX-like (LOXL) proteins, reinforce matrix stiffness. LOX-mediated crosslinking is shown to precede collagen deposition and accelerate fibrogenesis [11].

### 2.3. Limitations of Conventional Models

Standard 2D monocultures of hepatocytes or HSCs do not fully represent the in vivo system due to the lack of 3D architecture, cell–cell interactions, and ECM gradients characteristic of liver tissue. Another problem is the rapid loss of primary cell characteristics of hepatocytes in 2D culture over time, while the use of immortalized cell-lines may show variable metabolic outcomes. The 2D format also fails to capture chronicity, complex immune involvement, and gut–liver interactions [6]. Rodent models including bile duct ligation, or treatment with carbon tetrachloride or thioacetamide, have been explored as liver fibrosis models. However, they diverge from human pathophysiology. Differences in bile acid pools, metabolic regulation, immune composition, and regenerative capacity contribute to discrepancies between preclinical and clinical outcomes. These limitations have inspired the development of 3D human models to more accurately reconstruct multicellular, mechanobiological, and microbiome-modulated aspects of fibrosis [6].

## 3. Human Liver Models: Platforms and Readouts

### 3.1. Spheroids and Organoids

Multicellular spheroids and organoids represent the most accessible tier of 3D liver models, bridging the gap between traditional 2D monolayers and complex tissue-engineered constructs. These models are typically generated from primary human hepatocytes (PHHs), NPCs, or derived from induced pluripotent stem cells (iPSCs) [6]. Unlike simple clusters, self-organized organoids derived from iPSCs or adult progenitors recapitulate embryonic-like biliary and hepatic differentiation, forming polarized structures with functional bile canaliculi [6]. To model the fibrotic cascade, these systems incorporate hepatic stellate cells (HSCs), Kupffer cells (KCs), and liver sinusoidal endothelial cells (LSECs). When subjected to chronic injurious stimuli such as palmitic acid for MASLD modeling or TGF-β for direct HSC activation, these models exhibit significant ECM deposition and architectural remodeling [12].

These models are compatible with high throughput screening technologies like high content imaging. Second harmonic generation and two-photon microscopy can be used for the label-free quantification of fibrillar collagen. At the genomic level, high-throughput RNA-seq and spatial transcriptomics reveal the activation of fibrogenic genes (e.g., COL1A1, ACTA2). Similarly, multiplexed enzyme-linked immunosorbent assay (ELISA) is generally used for the measurement of secreted pro-inflammatory cytokines (e.g., IL-6, TNF-α) and matrix components (e.g., TIMP-1). Incomplete vascularization or lack of microfluidics, limited oxygen diffusion and nutrient transport, and protocol heterogeneity remain significant challenges in using organoids or spheroids, particularly for long-term, chronic injury models.

Liver assembloids build upon traditional spheroid and organoid concepts by combining and organizing various lineage-specific modules. These modules include hepatocyte-rich aggregates, cholangiocyte organoids, endothelial networks, and mesenchymal/immune clusters to create a more complex tissue that replicates specific anatomical structures. Instead of relying on random aggregation, assembloids are created by carefully arranging pre-formed organoids or cell aggregates. This is often done using micro-patterned scaffolds or bioprinted templates to establish consistent connections between parenchymal, biliary, stromal, and vascular compartments [13]. In a recent study on periportal liver assembloids, iPSC-derived hepatocyte-like cells were mixed with cholangiocyte organoids and portal fibroblast-like mesenchyme to form bile duct-like structures with patent lumina. These structures exhibited polarized transport, bile acid handling, and a transcriptomic profile closely aligned with human portal tracts [12]. When exposed to pro-inflammatory and pro-fibrotic signals (e.g., TNF, IL-4, IL-6, TGF-β), liver assembloids displayed hallmark features of biliary fibrosis. They activate NF-κB, JAK-STAT, and TGF-β pathways, and showed ductular reactions such as cholangiocyte expansion, peri-ductal collagen deposition, and portal fibroblast activation, closely resembling pathological signatures seen in patient biopsies of primary sclerosing cholangitis and primary biliary cholangitis [14]. Assembloids offer a controlled platform for precisely engineering cellular composition and spatial organization. By adjusting the abundance of portal fibroblasts, endothelial cells, Kupffer-like macrophages, and lymphocytes, researchers can study immune–stromal crosstalk, Notch-TGF-β signaling, cholangiocyte proliferation, and ECM remodeling during early fibrogenesis [13]. New technologies are working on integrating vascular elements such as patterned liver sinusoidal endothelial cells and perfusable channels into liver assembloids. These features recreate oxygen and nutrient gradients along with shear-dependent signals, allowing for zone-specific modeling of injury and a more accurate representation of pericentral versus periportal fibrosis trajectories in MASLD and other chronic liver diseases [13]. In general, liver assembloids that contain both hepatocytes and non-parenchymal cells across multiple lineages more accurately mimic periportal architecture. These assembloids demonstrate zonated periportal functions such as urea production, gluconeogenesis, biliary disease phenotypes, and personalized gene expression profiles. Recent studies have shown successful creation and replication of in vivo functionalities and disease modeling in mouse and human assembloids [13,14]. These findings indicate that their effectiveness surpasses that of traditional hepatocyte organoids, particularly in endothelial-integrated and periportal liver assembloid systems.

### 3.2. Bioprinted and Engineered Constructs

**Figure 2:**
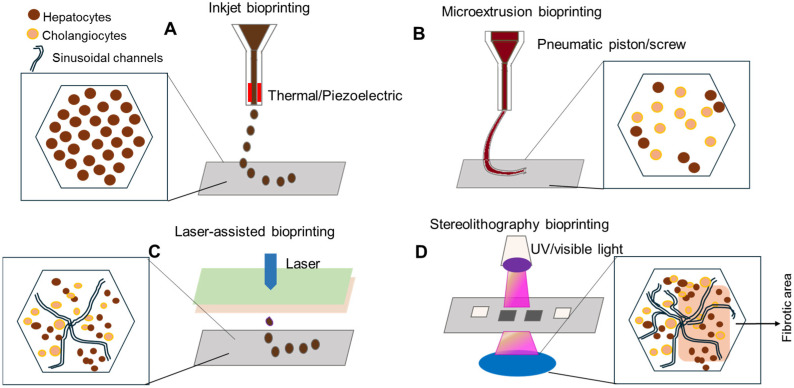
Overview of the major 3D bioprinting modalities. (**A**) Inkjet systems generate discrete droplets through thermal, electrostatic, or piezoelectric actuation. (**B**) Extrusion bioprinting dispenses continuous bioink filaments using pneumatic, piston, or screw-driven force. (**C**) Laser-assisted bioprinting propels bioink droplets through laser-induced microbubble formation. (**D**) Stereolithography bioprinting photopolymerizes bioresins layer-by-layer using patterned ultraviolet (UV) or visible light. This image was redrawn based on Lazaridou et al. [17].

3D bioprinting provides precise spatial control over the distribution of specific cell types and the composition of the ECM. By using bioinks made from decellularized liver ECM (dECM) or synthetic hydrogels with optimized stiffness, researchers can replicate the lobular or sinusoidal architecture of the human liver [15]. These engineered tissues are valuable for studying mechanotransduction, understanding how the increasing stiffness of a fibrotic matrix contributes to HSC myofibroblastic transformation. Bioprinted models allow for the controlled adjustment of matrix crosslinking, making them ideal for testing inhibitors of LOX and integrin signaling to explore new approaches to managing fibrogenesis (Figure 2) [16].

Current 3D bioprinting technologies include inkjet, laser-induced forward transfer (LIFT), and extrusion-based methods, each with bottleneck for liver constructs. Inkjet printing offers high speed, resolution (<50 μm), and cost-efficiency but requires low-viscosity bioinks that compromise structural integrity. Modifications like thrombin-crosslinked fibrin/collagen or saponified gelatin methacryloyl (GelMA) enhance stability while preserving viability [18]. LIFT excels in nozzle-free precision for high-viscosity inks and high cell densities, ideal for capillary networks. However, its high cost and slowness limit scalability. Extrusion-based printing dominates for versatility across bioinks, enabling heterogeneous lobule models with hepatocytes, endothelial cells, and sacrificial inks for lumens, as demonstrated by enhanced albumin/urea synthesis and Cytochrome P450 (CYP450) activity in patterned co-cultures [18]. Current liver organoid platforms use induced pluripotent stem cells (iPSCs), human embryonic stem cells (hESCs), and HepaRG lines. iPSCs are particularly valued for patient-specific differentiation into hepatocytes, stellate cells, and cholangiocytes, though there are challenges such as teratoma risk and scalability. Biofabrication strategies employ multi-component hydrogels (GelMA-dECM, alginate-methylcellulose-Matrigel) to achieve shear-thinning, rapid gelation, and ECM mimicry, while scaffold-free spheroid or Kenzan fusion approaches enhance self-ECM secretion but lack mechanical stability without stromal support [18,19]. Recent advances highlight parenchymal models such as HepaRG-derived hepatorganoids with microvascularization, alongside vascular innovations like angiogenic gradients between perfusable channels, leading towards functional integration of liver assembloids [20]. Although bioprinted constructs offer high structural accurately, they currently require specialized biofabrication equipment, experience batch-to-batch variations in bioink properties, and need further optimization. Additionally, they encounter challenges related to maintaining long-term metabolic zonation without continuous perfusion and immune integration. Future prospects for liver bioprinting depend on mitigating ischemic stress during fabrication, a challenge that can be addressed through the use of perfused scaffolds or segmented printing strategies. In parallel, efforts to optimize biliary function involve the use of stereolithography with composite matrices such as collagen, hyaluronic acid, fibrinogen, and Matrigel to restore epithelial polarity in cholangiocytes. Bioreactor systems like FABRICA provide real-time oxygenation and monitoring during scaffold-free printing, ultimately enhancing tissue viability [21]. Applications in disease modeling, including fibrosis spheroids, and drug screening platforms underscore the translational potential of these constructs. With ongoing advancement in vascular and biliary bioprinting, along with improved iPSC maturation protocols, 3D bioprinted livers show promise in eliminating the need for immunosuppression. This transformation could revolutionize therapy for chronic liver disease and provide efficient models for fibrosis drug discovery.

A key issue in bioprinted and other dense 3D fibrotic constructs is how ECM organization shapes drug penetration and local pharmacokinetics. Increasing collagen deposition, LOX-driven crosslinking, and proteoglycan buildup can sharply restrict diffusion and generate steep concentration gradients from the surface to the core [22]. Observed drug inactivity may therefore reflect poor tissue penetration rather than a true lack of potency. Pairing functional assays with tracer-based measurements using fluorescent probes, labeled biologics, or mass spectrometry imaging, and, when possible, incorporating diffusion-reaction modeling, may help to distinguish these effects. Such integrated pharmacokinetic assessments help ensure that negative findings arise from genuine pharmacodynamic limitations rather than ECM-mediated shielding [23].

### 3.3. Liver-on-a-Chip and Microphysiological Systems (MPSs)

Liver-on-chip platforms are currently the main focus under New Approach Methodologies (NAMs) regulations. These platforms involve embedding human liver cells within microfluidic devices that provide controlled perfusion and physiological shear stress [24]. These systems are uniquely capable of reconstructing the liver sinusoid, with LSECs form a barrier that regulates the interaction between circulating immune cells and underlying hepatocytes [25]. Some major advantages of similar microphysiological systems (MPSs) models in fibrosis research and drug discovery include: (1) the potential to represent zonation, where flow-induced oxygen gradients allow for the study of zone-specific fibrosis, typically occurring first in the pericentral region (Zone 3); (2) the dynamic stimulation potential enabling the chronic, pulsatile delivery of drugs or nutrients, better mimicking the human post-prandial metabolic cycle compared to static cultures; and (3) integrated sensors that can measure Trans-Epithelial Electrical Resistance (TEER) to monitor endothelial dysfunction in real-time.

The choice of platform depends on the specific “Context of Use” (CoU) within the drug development pipeline. Spheroids and organoids are optimized for high-throughput screening (HTS) and toxicity ranking (Table 1). In contrast, bioprinted tissues and liver-on-chip systems provide the mechanistic depth required for lead optimization and the de-risking of anti-fibrotic candidates prior to clinical entry.

Emerging technologies combine microfluidics with high-speed imaging to quantify mechanical, strain which can be translated to the fibrotic stage [26]. Recently a co-culture model of endothelial and fibroblast spheroids was reported, revealing composition-dependent mechanical differences. Treatment with TGF-β1 and the anti-fibrotic drug Nintedanib demonstrated how cell–cell and cell–ECM interactions shape spheroid mechanics [27]. These findings link mechanical properties to fibrosis progression and position the platform as a promising tool for disease modeling and anti-fibrotic drug development. In current fibrosis models, one major limitation is the minimal representation of adaptive immunity. Most of these models lack bona fide T or B cells, making them less capable of studying autoimmune hepatitis and cholangiopathies with autoreactive T cells. Further research and development may lead to better liver fibrosis models incorporating mature adaptive immune components to fully mimic T-cell driven fibrosis in vivo. Even though recent models contain HSCs and macrophages, the adaptive responses like Th-17 IL-17 secretion and Treg modulation are absent, indicating the need for further progress and development [27].

Beyond the shared characteristics of the 3D liver models, different 3D platforms are particularly well-suited for modeling distinct stages of liver fibrosis. Multicellular spheroids and organoids are effective for examining early hepatocyte injury, HSC activation, and the onset of extracellular matrix deposition under defined metabolic or inflammatory cues, consistent with reports showing their utility in capturing early fibrogenic transitions [3]. Liver assembloids, which incorporate spatially organized epithelial, mesenchymal, and immune niches, enable the analysis of zone-specific fibrogenesis, including periportal versus pericentral injury patterns, ductular reactions, and portal fibroblast activation reflecting observations from recent spatially resolved liver models [4]. Bioprinted constructs, with tunable gradients of stiffness and matrix composition, are well positioned to reproduce later-stage architectural distortion, bridging septa, and the mechanical consequences of dense scar tissue, as demonstrated in engineered fibrosis tissues with controlled ECM mechanics [4]. Liver-on-chip and other microphysiological systems (MPSs) integrate perfusion, zonation cues, and multi-organ connectivity, making them uniquely suited for modeling dynamic progression and regression of fibrosis and for incorporating pharmacokinetic and pharmacodynamic considerations such as repeated dosing and systemic crosstalk [5]. Selecting a model that aligns with the specific stage of fibrosis under investigation is therefore key for designing informative and translationally relevant preclinical studies.

### 3.4. Advanced Animal Models as Benchmarks for Human-Relevant Systems

Even though this review focuses on human-relevant systems, advanced in vivo models remain important as validation and benchmarking tools. Contemporary murine models of liver fibrosis include chemically induced injury (such as CCl_4_ or TAA), diet-based regimens (Western diet, choline-deficient high-fat diet, or methionine- and choline-deficient), surgical interventions like bile duct ligation, as well as genetic and immune-mediated systems. Each category captures distinct histopathologic and immunologic aspects of human fibrotic liver disease [28]. Increasingly, investigators employ combination models that layer dietary stressors onto toxic exposures or specific genetic backgrounds to better mimic MASLD/MASH-related fibrosis [29]. These hybrid approaches also allow fibrosis to regress once the damaging stimulus is removed, providing a powerful framework for dissecting extracellular matrix turnover, HSC inactivation, and macrophage phenotype transitions in vivo.

Next-generation humanized and chimeric liver mouse models extend these experimental frameworks by enabling engraftment of functional human hepatocytes and, increasingly, non-parenchymal cell populations involved in chronic injury [30]. This capacity creates a versatile platform for assessing human-specific drug metabolism, immunopathologic responses, and toxicity within a fibrotic milieu. When combined thoughtfully with organoids, bioprinted liver constructs, and liver-on-chip systems, these in vivo models function as critical benchmarks for cross-species alignment. They facilitate calibration of histologic, biomechanical, and transcriptomic fibrosis metrics; allow rigorous evaluation of anti-fibrotic agents under systemic and immunocompetent conditions; and support preclinical validation of non-invasive biomarkers prior to their translation into clinical studies.

### 3.5. Metabolic Competence in Fibrosis Models

For anti-fibrotic drug development, the metabolic maturity of liver model systems remains a central determinant of their predictive performance. Primary human hepatocytes (PHHs) continue to serve as the benchmark for xenobiotic metabolism because they retain physiologic levels of phase I and phase II enzymes as well as key uptake and efflux transporters. However, they are limited by donor variability, rapid loss of phenotype in standard 2D culture, and restricted long-term function [31]. Spheroids, organoids, and microphysiological systems can recover and prolong aspects of PHH activity, but each platform still requires thorough assessment of CYP450 function, conjugation pathways, and transporter expression. In contrast, iPSC-derived hepatocyte-like cells and many hepatic progenitor models frequently exhibit a fetal-like state, characterized by diminished CYP isoform expression, perturbed nuclear receptor signaling, and immature bile acid metabolism. HepG2 and similar lines provide more consistent, though still incomplete, metabolic competence [32].

From a development standpoint, inadequate metabolic capacity can produce false-negative outcomes, especially for prodrugs or compounds that depend on hepatic activation or extensive biotransformation to achieve anti-fibrotic activity. Conversely, exaggerated or non-physiologic metabolism may inflate toxicity estimates [33]. Recommended practices therefore include (i) benchmarking CYP450 and conjugating enzyme activities (e.g., CYP3A4, CYP2C9) against PHH standards, (ii) tracking major nuclear receptor pathways such as CAR, and (iii) integrating physiological flow, co-culture, and zonation cues to promote maturation. Newer approaches including transcription factor-driven programming, micropatterned co-cultures with stromal partners, and extended perfusion in liver-on-chip devices are helping narrow the metabolic maturity gap [34]. Fibrotic models may require careful validation of their metabolic competence before being used for quantitative ranking of candidate therapeutics.

## 4. Microbiome-Integrated Liver Models

Advancements in tissue engineering have made it possible to create sophisticated microbiome-integrated MPSs. A key approach involves enhancing liver organoids or liver-on-chip systems with physiological levels of microbial metabolites, such as short-chain fatty acids (SCFAs), indoles, and bile acids, to investigate their specific roles in HSC activation [35]. Advanced MPS models use connected gut–liver platforms, where an intestinal epithelium channel is fluidically linked to a downstream liver compartment. These systems enable exposure to live microbial communities at the apical side while directing effluent to the liver tissue at the basolateral side under controlled perfusion. These personalized models, often incorporating patient-derived organoids, offer a strong foundation for studying disease-specific fibrogenesis and predicting individual responses to microbiome-targeted treatments [36]. One of the major factors for an effective microbiome-integrated MPS platform is the ability to sustain bacterial growth without experiencing overgrowth or sepsis. The usage of semi-permeable membranes or gel barriers to separate bacteria from the MPS systems while still allowing microbial metabolites is an efficient system. The inclusion of fluidics, real-time monitoring of oxygen, pH, and barrier integrity can help to track microbial growth and facilitate long-term co-culturing [37]. Recent findings have identified certain taxa, like *Akkermansia muciniphila*, as protective agents that enhance barrier function and regulate hepatic immune response through metabolites like propionate [38]. These findings emphasize the importance of incorporating microbiome-relevant signals into liver models that accurately reflect disease mechanisms. Microbiome signatures, including specific taxa and diversity measures, are increasingly used as predictive biomarkers for liver fibrosis staging. Integrative multi-omics studies have connected these microbial patterns to the severity of MASLD and alcohol-associated liver disease (ALD). These 3D models provide a framework for validating these signatures and assessing the effectiveness of emerging anti-fibrotic treatments (Table 2).

Emerging evidence indicates that modulation of the microbiome can attenuate liver fibrosis, with preclinical and early clinical studies reporting disease mitigation [40]. Probiotics and synbiotics have been shown to reduce liver stiffness measurement (LSM) and controlled attenuation parameter (CAP) scores in patients with MASLD, outperforming placebo by improving steatosis and fibrosis markers [40]. Fecal microbiota transplantation (FMT) has been shown to restore microbial diversity, reduce Proteobacteria abundance, and attenuate HSC activation by reshaping the intrahepatic immune microenvironment, as demonstrated in animal models and pilot human trials of cirrhosis and hepatic encephalopathy (HE) [6]. Multi-omics validation in coupled gut–liver MPS further confirms that these interventions suppress fibrogenic pathways, with autologous FMT exhibiting the greatest efficacy in reducing hepatic lipid accumulation [40,41]. These platforms also reveal modulation of bile acid metabolism and short-chain fatty acid (SCFA) production, underscoring how microbial metabolites shape hepatocyte stress responses and ECM remodeling [41]. Collectively, such integrated systems offer translational validation that microbiome modulation can directly influence the progression of liver fibrosis. However, differences in the diagnostic approaches of liver fibrosis such as biopsy and variable non-invasive tests, along with the limited characterization of intestinal microbiome, may lead to inconsistent findings across studies [42]. Emphasis on unified sample collection methods, sequencing platforms, and bioinformatic pipelines is needed to produce consistent microbiome profiles [43]. Collectively, these methodological and biological sources of variability need to be considered for future microbiome–liver disease studies.

## 5. Biomarkers and Therapeutic Modalities

Biomarker discovery is crucial for advancing anti-fibrotic therapy because it enables patient stratification, early detection, and monitoring of therapeutic response. Serum markers such as hyaluronic acid, procollagen type III N-terminal peptide (PIIINP), and tissue inhibitor of metalloproteinases-1 (TIMP-1) have been validated as part of composite indices like the Enhanced Liver Fibrosis (ELF) score, which strongly correlates with histological fibrosis stage [44]. Imaging biomarkers like transient elastography (FibroScan) and magnetic resonance elastography offer non-invasive quantification of liver stiffness and are increasingly used in clinical trials as surrogate endpoints [45]. These methods reduce the need for biopsy and provide reliable patient-friendly alternatives for long-term monitoring. The translational validity of 3D liver models depends on their ability to reflect these clinical endpoints. Specifically, human liver MASH models have shown the ability to secrete clinical biomarkers such as TIMP-1, with its levels particularly upregulated in a disease state [44]. Furthermore, the physical properties of these constructs, such as their compressive modulus or stiffness, can be quantified using micro-indentation or atomic force microscopy to provide a direct in vitro correlate to clinical FibroScan data, allowing for the longitudinal assessment of scaffold remodeling in response to anti-fibrotic agents [46].

Proteomic and metabolomic profiling of plasma and stool samples further emphasize the role of gut–liver axis metabolites, such as bile acids and short-chain fatty acids, in the progression of fibrosis. By combining these biomarkers with genetic risk factors (*PNPLA3*, *TM6SF2*) into predictive algorithms, precision medicine approaches can be developed to customize anti-fibrotic interventions for individual patients. These biomarker-driven strategies have the potential to accelerate drug development pipelines and improve clinical outcomes. Yuan and colleagues used patient-specific human hepatocyte organoids and mesenchymal cells to generate liver assembloids. This approach enabled the validation of multi-omic signatures in a controlled environment while preserving the unique genetic background of the patient [13].

A biomarker-driven therapeutic strategy for hepatic fibrosis should integrate targeted small molecules, biologics, nucleic-acid modalities and microbiome-directed interventions to interrupt the core fibrogenic circuits identified in regenerative and injury studies. Small-molecule inhibitors that attenuate TGF-β signaling and PDGF-driven stellate cell activation remain central to reducing ECM deposition and myofibroblastic conversion [47]. These approaches can be combined with LOXL2 inhibitors or other agents that limit collagen crosslinking to both prevent new matrix accumulation and soften established scar tissue, thereby improving scaffold remodeling and cell engraftment [10]. Complementary biologic strategies like neutralizing monoclonal antibodies and receptor-Fc fusion proteins can be deployed to sequester pro-fibrotic cytokines during the priming and proliferation phases of regeneration. The cytokine candidates can be identified in systematic cytokine screens, while nanoparticle-enabled delivery improves tissue targeting and reduces off-target exposure [48]. Finally, HSC-targeted siRNA and peptide/nanoparticle platforms provide a practical route to silence fibrogenic transcripts in situ and have demonstrated efficacy in preclinical HSC-targeting studies [49].

A recent review by Codotto et al. highlights how single-cell and spatial transcriptomics, along with proteomic and metabolomic profiling, are redefining biomarker discovery by mapping cell-type-specific injury responses and fibrotic niches [7]. The article also emphasizes the value of iPSC-derived liver models and organoids as patient-specific systems for validating candidate biomarkers and predicting therapeutic responses [7]. The integration of assembloid readouts, such as quantifying soluble TIMP-1 and measuring tissue stiffness, along with insights from multi-omics and genetic risk factors, is expected to bridge the gap between benchtop models and clinical fibrosis staging. This integration will ultimately accelerate precision medicine strategies [50].

## 6. Regulatory Qualification and Translational Outlook

The regulatory landscape is shifting toward the acceptance of NAMs as complementary tools for drug safety and efficacy assessment. The FDA’s Innovative Science and Technology Approaches for New Drugs (ISTAND) pilot program has already accepted an organ-on-a-chip Liver-Chip submission for DILI assessment [51], illustrating a clear regulatory pathway for qualified microphysiological systems. For these 3D liver models to achieve widespread adoption, rigorous characterization of cell identity, zonation, and quantitative benchmarking against human in vivo reference data is essential. Recent reviews emphasize the need for standardized metrics and combined organoid with liver-on-chip strategies to capture multicellular crosstalk and gut–liver interactions relevant to anti-fibrotic mechanisms [52].

A practical way to standardize the evaluation of 3D human liver fibrosis models Is to define a core set of validation criteria that can be adapted to different experimental goals. At the molecular level, models should show clear and reproducible induction of well-established fibrogenic genes, including collagen type I (COL1A1) and α-smooth muscle actin (ACTA2), along with appropriate changes in cytokines and matrix-modifying factors. This approach is consistent with recent reports emphasizing molecular confirmation of fibrotic activation in engineered systems [23]. Morphologically, the presence and organization of fibrillar collagen should be verified using second-harmonic generation or comparable imaging methods, with quantitative assessment of fiber abundance and architecture, as recommended in current 3D fibrosis modeling studies. Functionally, constructs should demonstrate increased matrix stiffness measured by techniques such as atomic force microscopy, indentation, or optical elastography and may also show elevated secretion of extracellular matrix proteins like TIMP-1. In more advanced stages, a decline in hepatocyte function, reflected by reduced albumin or urea output, provides additional confirmation of disease-relevant impairment, a pattern observed in validated bioprinted and drug-induced fibrosis models. Considering molecular, structural, and functional readouts together offers a practical framework for determining when a 3D system authentically reflects a fibrotic phenotype and supports meaningful comparison across platforms and laboratories [46].

As qualification frameworks mature, next-generation liver MPS platforms are poised to transform anti-fibrotic drug discovery by enabling mechanistic, patient-relevant modeling that traditional animal systems cannot capture. Multi-cellular Liver-Chip systems incorporating hepatocytes, Kupffer cells, stellate cells, and endothelial cells already demonstrate improved prediction of hepatotoxicity and fibrogenic responses, aligning with regulatory expectations for human-relevant performance standards [4]. Integration of liver models with gut, vascular, or immune modules will further enhance translational fidelity by capturing inter-organ signaling pathways including bile acid flux, microbial metabolite transfer, and macrophage-HSC crosstalk that drive fibrosis progression [53]. As reproducibility improves and standardized qualification metrics emerge, these platforms are expected to support biomarker validation, dose optimization, and early de-risking of anti-fibrotic candidates, accelerating the path from discovery to clinical translation. Ultimately, the convergence of regulatory acceptance and technological maturity positions MPS as a cornerstone of future anti-fibrotic pipelines.

Despite challenges related to reproducibility and long-term phenotypic stability, the strategic combination of organoids and liver-on-chip platforms or similar technologies may transform anti-fibrotic development by providing more predictive, human-relevant models that accelerate translation from bench to clinic [4,50].

## Figures and Tables

**Table 1:** Comparison of selected complex in vitro models for modeling liver fibrosis.

Feature	Spheroids & Organoids	Bioprinted Constructs	Liver-on-a-Chip (MPS)
Cellular Organization	Self-organized clusters (Spherical)	Controlled spatial placement (Lobular)	Micro-engineered layers (Sinusoidal)
Physiological Fidelity	Moderate (Metabolic activity)	High (Structural/Mechanical)	Highest (Dynamic/Perfusion)
Throughput	High (96/384-well compatible)	Low to Moderate	Low
Mechanical Cues	Limited (Uniform stiffness)	High (Tunable ECM stiffness)	Moderate (Shear stress focus)
Vascularization	None (Diffusion-limited)	Potential (Pre-printed channels)	High (Functional lumen/flow)
Primary Strength	Scalability for HTS/Discovery	Mechanobiology and LOX studies	PK/PD and Multi-organ crosstalk
Primary Limitation	Lacks perfusion/zonation	Technically demanding; bioink variability	High cost; low throughput
Readout Capability	Bulk omics, imaging	Spatial omics, stiffness mapping	Real-time sensing (TEER, oxygen)

Abbreviations used: ECM, extracellular matrix; HTS, high-throughput screening; LOX, lysyl oxidase; MPS, microphysiological system; PK/PD, pharmacokinetic and pharmacodynamics; TEER, trans-epithelial electrical resistance.

**Table 2:** Gut microbial taxa implicated in liver fibrosis and their proposed mechanisms.

Microbe/Taxon	Impact on Liver Fibrosis	Evidence in Liver Disease	Proposed Mechanism(s)
*Enterobacteriaceae (family)*	Pro-fibrotic	Enriched in HBV/HCV, cirrhosis, ALD	LPS endotoxemia, TLR4 activation on Kupffer/HSCs, stellate cell activation
*Veillonellaceae (family)*	Pro-fibrotic	Increased in HBV/HCV cirrhosis cohorts	Dysbiosis contribution, lactate fermentation, systemic inflammation
*Bifidobacterium* spp.	Anti-fibrotic/protective	Reduced in chronic HBV/HCV, cirrhosis	SCFA production, barrier reinforcement, reduced translocation/endotoxemia
*Lactobacillus* spp.	Anti-fibrotic/protective	Decreased in HBV/HCV cirrhosis; probiotic target	Anti-inflammatory cytokines, bile acid modulation, epithelial integrity
*Akkermansia muciniphila*	Anti-fibrotic/protective	Depleted in NASH/cirrhosis; protective enrichment	Mucin remodeling, gut barrier enhancement, lower portal LPS
*Faecalibacterium prausnitzii*	Anti-fibrotic/protective	Reduced in HBV cirrhosis, advanced fibrosis	Butyrate/SCFA production, anti-inflammatory, barrier support

Data was compiled from [39]. Abbreviations used: ALD, alcohol-associated liver disease; HBV, hepatitis B virus; HCV, hepatitis C virus; LPS, lipopolysaccharide; SCFA, short-chain fatty acid; TLR, toll-like receptor

## Data Availability

No new data were created or analyzed in this study. Data sharing is not applicable to this article.

## Abbreviations

The following abbreviations are used in this manuscript:

2D	Two-dimensional
3D	Three-dimensional
ALD	Alcoholic liver disease
AI	Artificial intelligence
BMP(s)	Bone morphogenetic proteins
CAP	Controlled attenuation parameter
CAR	Constitutive Androstane Receptor
CoU	Context of Use
CYP450	Cytochrome P450
CYP3A4	Cytochrome P450 Family 3 Subfamily A Member 4
CYP2C9	Cytochrome P450 Family 2 Subfamily C Member 9
dECM	Decellularized liver ECM
DILI	Drug-induced liver injura
ECM	Extracellular matrix
ELF	Enhanced liver fibrosis
ELISA	Enzyme-linked immunosorbent assay
EU	European Union
FDA	Food and Drug Administration
GDF(s)	Growth differentiation factor(s)
GLP-1	Glucagon-like peptide-1
HBV	Hepatitis B virus
HCC	Hepatocellular carcinoma
HCV	Hepatitis C virus
HESC(s)	Human embryonic stem cell(s)
IL-1β	Interleukin-1 beta
IL-4	Interleukin-4
IL-6	Interleukin-6
IL-10	Interleukin-10
IL-17	Interleukin-17
iPSC(s)	Induced pluripotent stem cell(s)
ISTAND	Innovative Science and Technology Approaches for New Drugs
EILSA	Enzyme-linked immunosorbent assay
FMT	Fecal microbiota transplantation
GelMA	Gelatin methacryloyl
HE	Hepatic encephalopathy
HSC(s)	Hepatic stellate cell(s)
HTS	High-throughput screening
JAK-STAT	Janus kinase/signal transducer and activator of transcription
KC(s)	Kupffer cell(s)
LIFT	Laser-induced forward transfer
LOX	Lysyl oxidase
LOXL	Lysyl oxidase-like
LPS	Lipopolysaccharide
LSEC(s)	Liver sinusoidal epithelial cell(s)
LSM	Liver stiffness measurement
MASH	Metabolic dysfunction-associated steatohepatitis
MASLD	Metabolic dysfunction-associated steatohepatitis
NAFLD	Non-alcoholic fatty liver disease
NASH	Non-alcoholic steatohepatitis
MPS(s)	Microphysiological system(s)
NAM(s)	New Approach Methodology(s)
NF-κB	Nuclear factor kappa B
NK	Natural killer
NKG2D	NK group 2 member D (activating receptor)
NPC(s)	Non-parenchymal cell(s)
PHH(s)	Primary human hepatocyte(s)
PDGF	Platelet-derived growth factor
PK/PD	Pharmacokinetics/pharmacodynamics
PSC	Primary sclerosing cholangitis
RNA-seq	RNA sequencing
SCFA(s)	Short-chain fatty acids
TEER	Trans-epithelial electrical resistance
TGF-β	Transforming growth factor-β
Th1	T helper 1
TH17	T helper 17
THR-β	Thyroid hormone receptor-beta
TIMP-1	Tissue inhibitor of metalloproteinase-1
TLR	Toll-like receptor
TNF-α	Tumor necrosis factor-α
TRAIL	TNF-related apoptosis-inducing ligand
Treg	Regulatory T cell
UV	Ultraviolet
